# Estimating the total prevalence and incidence of end-stage kidney disease among Aboriginal and non-Aboriginal populations in the Northern Territory of Australia, using multiple data sources

**DOI:** 10.1186/s12882-017-0791-3

**Published:** 2018-01-15

**Authors:** Lin Li, Steven Guthridge, Shu Qin Li, Yuejen Zhao, Paul Lawton, Alan Cass

**Affiliations:** 1 Northern Territory Department of Health, Health Gains Planning, Darwin, NT Australia; 2 Menzies School of Health Research, Charles Darwin University, Darwin, NT Australia

**Keywords:** End stage kidney disease, Renal replacement therapy, Capture–recapture method, Prevalence, Incidence, Data linkage

## Abstract

**Background:**

Most estimates for End Stage Kidney Disease (ESKD) prevalence and incidence are based on renal replacement therapy (RRT) registers. However, not all people with ESKD will commence RRT and estimates based only on RRT registry data will underestimate the true burden of ESKD in the community. This study estimates the total number of Northern Territory (NT) residents with ESKD including: those receiving RRT, those diagnosed but not receiving RRT and an estimate of “undiagnosed” cases.

**Methods:**

Four data sources were used to identify NT residents with a diagnosis of ESKD: public hospital admissions, Australia and New Zealand Dialysis and Transplant Registry registrations, death registrations and, for the Aboriginal population only, electronic primary care records. Three data sources contained information recorded between 1 July 2008 and 31 December 2013, death registration data extended to 31 December 2014 to capture 2013 prevalent cases. A capture–recapture method was used to estimate both diagnosed and undiagnosed cases by making use of probability patterns of overlapping multiple data sources.

**Results:**

In 2013, the estimated ESKD prevalence in the NT Aboriginal population was 11.01 (95% confidence interval (CI) 10.24–11.78) per 1000, and 0.90 (95% CI 0.76–1.05) per 1000 in the NT non-Aboriginal population. The age-adjusted rates were 17.97 (95% CI 17.82–18.11) and 1.07 (95% CI 1.05–1.09) per 1000 in the NT Aboriginal and non-Aboriginal populations respectively. The proportion of individuals receiving RRT was 71.4% of Aboriginal and 75.5% of non-Aboriginal prevalent ESKD cases. The age-adjusted ESKD incidence was also greater for the Aboriginal (5.26 (95% CI 4.44–6.08) per 1000 population) than non-Aboriginal population (0.36 (95% CI 0.25–0.47) per 1000).

**Conclusion:**

This study provides comprehensive estimates of the burden of ESKD including those cases that are not identified in relevant health data sources. The results are important for informing strategies to reduce the total burden of ESKD and to manage the potential unmet demand, particularly from comparatively young Aboriginal patients who may be suitable for RRT but do not currently access the services for social, geographic or cultural reasons.

**Electronic supplementary material:**

The online version of this article (doi: 10.1186/s12882-017-0791-3) contains supplementary material, which is available to authorized users.

## Background

End-stage kidney disease (ESKD) is a significant and growing public health problem, associated with high morbidity, mortality and diminished quality of life. ESKD also generates disproportionately high costs to the health care system as patients require renal replacement therapy (RRT), by dialysis or kidney transplantation, for long-term survival [1]. The Northern Territory (NT) is a federal territory of Australia occupying much of the central and northern part of the country and has a much greater proportion of Aboriginal Australians (30%) than the balance of Australia (3%). The NT has the highest incidence and prevalence of RRT for ESKD among all states and territories in Australia [2]. The incidence of RRT among Aboriginal Australians is substantially higher than non-Aboriginal Australians, and among Aboriginal Australians the incidence of RRT is highest in the NT [2].

A limitation for international reporting of the prevalence and incidence of ESKD is that estimates are generally restricted to individuals who receive RRT, as recorded and reported by national or regional data registries [3]. In Australia, the Australia and New Zealand Dialysis and Transplant Registry (ANZDATA) reports individuals who receive RRT [2]. This approach will underestimate the true burden of ESKD in the community [1], missing individuals with diagnosed ESKD but not accessing RRT as well as an uncertain number of undiagnosed cases. One previous Australian study estimated total ESKD incidence by linking ANZDATA data with national mortality data and reported an age-standardised incidence rate, for 2003–2007, for the total NT population of 0.72 per 1000, much higher than the corresponding national rate (0.2 per 1000) [4]. A separate study, also linked ANZDATA data and national mortality data and reported that for 2009–2013, the age-standardised incidence rate for ESKD was almost five times higher in the Australian Aboriginal population than the non-Aboriginal Australian population (0.95 vs 0.19 per 1000 population) [1]. To our knowledge, there have been no studies that have estimated total ESKD prevalence in Australia.

The capture-recapture method was originally used in ecological research to quantify populations [5]. This method is now used in epidemiology to estimate ascertainment level by linking unit level data across multiple data sources [6, 7]. The capture–recapture method uses the degree of overlap of individuals between sources to estimate the number of undiagnosed cases not registered in any of the sources and hence provides a more complete estimate of the total number of cases in a population [8, 9]. This approach is particularly relevant for the Aboriginal population for whom anecdotal reports suggest a significant proportion may not be accessing services and as a result have conditions which remain undiagnosed.

The aim of this study was to estimate the total prevalence and incidence of ESKD in NT Aboriginal and non-Aboriginal populations, to examine the demographic characteristics of patients with ESKD, and the proportion of patients with ESKD who did not receive RRT.

## Methods

### Data sources

Four data sources were used to identify all individuals with a diagnosis of ESKD during the study period from 1 July 2008 to 31st December 2013. The NT Hospital Separations Dataset (HSD) contains administrative information on all admissions to all five NT public hospitals. The Primary Care Information System (PCIS) contains electronic clinical records for more than 30,000 Aboriginal patients who attend any of the 50 government-run primary health care centres located in remote NT communities. This is nearly half the total NT Aboriginal population. ANZDATA collates RRT data on all patients throughout Australia and New Zealand who receive maintenance dialysis or a kidney transplant [1, 2]. The fourth data source was death registrations from the NT Registry of Births, Deaths and Marriages (BDM), with available data extended to 31 December 2014. As PCIS data is largely confined to Aboriginal patients, only three data sources were used for non-Aboriginal estimates. Demographic characteristics of age, sex and Aboriginal status are shared between the health datasets and have been reported as highly reliable [10, 11]. Death registration data has recorded Aboriginal status, and has been combined with health data in many previous studies [12–14].

### Case definition

ESKD cases in HSD were identified using diagnosis codes which had been coded using the International Classification of Disease version 10, Australian Modification 6th edition (ICD-10-AM). The codes were selected based on previous work by the Australian Institute of Health and Welfare (AIHW) [15–17]. The definition for inclusion was a primary or secondary diagnosis code for N18.5, T82.4, T86.1, Z49, Z94.0 or Z99.2. ESKD cases in PCIS data were identified based on either: a diagnosis (coded using the International Classification of Primary Care (ICPC), 2nd edition) of stage 5 chronic kidney disease, dialysis or a renal transplant [18]; or laboratory data to estimate a glomerular filtration rate (eGFR) <15 mL/min/1.73m^2^ with previous eGFR <60 mL/min/1.73m^2^ at least 3 months prior [19]. Only NT residents were included in the study. For death registration data, a key word search was conducted to identify those individuals with either a leading cause of death recorded as dialysis, kidney (renal) transplant, end-stage kidney (renal) disease, end-stage kidney (renal) failure or chronic kidney (renal) failure, or the other causes of death having end-stage kidney (renal) failure. The definition was again based on existing AIHW methodology [15, 17]. Full details of the definitions for ESKD cases, including ICD-10-AM and ICPC codes are presented in Additional file 1: Table S1.

A sensitivity analysis was performed that examined the effect of different eGFR cut-off definitions of ESKD (<15 or = <7 mL/min/1.73m^2^) on the estimate for undiagnosed individuals in the model.

### Linkage of individuals in multiple datasets

The NT Department of Health uses a unique health identifier, known as the Hospital Registration Number (HRN), for all client services. The HRN has been used in a number of previous linkage studies and is highly reliable [11]. For this study the HRN was used for linkage of individual records in the three clinical datasets. Names, date of birth, sex, date of death and address were used to match death registration records and HRN. HRNs were then used to link death data with the other three datasets.

### Statistical analysis

For the NT Aboriginal population, ESKD cases identified in one or more of the four data sources were used for incidence and prevalence estimates. We used log-linear capture–recapture models, in which the degree of overlap between known cases in different datasets can be used to estimate the number of undiagnosed (unknown) cases in the population [9]. The log-linear model involves two-way interactions terms between data sources and covariates to adjust for interdependence between data sources. Separate models were undertaken for Aboriginal and non- Aboriginal populations. A *p*-value <0.05 was used as a cut-off for main effects and interactions. Dates of death were used to censor cases. The frequency of patients in the various combinations of datasets and the estimate of undiagnosed cases are presented in Additional files 2, 3, 4 and 5: Tables S2–S5.

Point prevalence was based on 31 December 2013. ESKD death records in 2014 were included in the prevalence model based on the assumption that if chronic kidney failure was sufficiently severe to be the leading cause of death, then it was likely to be ESKD, therefore those who died within the year after 31 December 2013 were included as cases in prevalence estimation. Incidence was calculated using all new diagnoses recorded in any of the data sources during 2013. We improved the reliability of identification of new cases in 2013 by using a 4.5 years washout period (01/07/2008–31/12/2012). ESKD cases in death registration data who died during 2013 but not found in the other three clinical datasets were included in the incidence model. For those cases untreated with RRT, year of death was used as a proxy for incidence year [15]. Prevalence and incidence of ESKD were estimated with and without undiagnosed cases. The primary source of information about Aboriginal status, age, and sex were from HSD, followed by ANZDATA, PCIS and BDM. Australian Bureau of Statistics estimates of NT resident populations were used as denominators for age specific rate calculations [20]. To accommodate age structure differences, the NT rates were directly age-standardised to the 2001 Australian population which came from the Standard Population for Use in Age-Standardisation produced by the Australian Bureau of Statistics [21]. The underestimation is calculated from the diagnosed cases divided by diagnosed cases plus undiagnosed cases which are generated from capture-recapture method.

### Ethics

The study protocol (HREC 2014-2211) was approved by the Human Research Ethics Committee of Northern Territory Department of Health and Menzies School of Health Research (EC 00153).

## Results

### Prevalence

On 31 December 2013, there were 814 known ESKD cases identified from at least one of the data sources (Table 1). Of known ESKD cases, 682 (83.8%) were Aboriginal people. The male: female sex ratio was in the opposite direction in Aboriginal (0.65) than non-Aboriginal (1.4) patients with known ESKD. The proportion of known ESKD cases treated with RRT was higher in non-Aboriginal (88.6%) than Aboriginal patients (82.0%) (Table 2, *P* < 0.05). Aboriginal patients were generally younger, with a median age of 53 years compared with 55 years for non-Aboriginal patients.

**Table 1:** Age distribution of known end-stage kidney disease cases by sex and Aboriginal status, Northern Territory, based on 31/12/2013

Age group	Aboriginal				Non-Aboriginal				Total
	Female	Male	Sub-total	%	Female	Male	Sub-total	%	
<35	22	17	39	5.7	8	12	20	15.2	59
35–44	62	47	109	15.9	8	6	14	10.6	123
45–54	127	92	219	32.0	11	16	27	20.5	246
55–64	135	80	215	31.6	14	15	29	22.0	244
65–74	57	23	80	11.8	14	20	34	25.8	114
>74	10	10	20	2.9	1	7	8	6.1	28
Total	413	269	682	100.0	56	76	132	100.0	814

**Table 2:** Prevalence of end-stage kidney disease by age group, sex and Aboriginal status, Northern Territory, based on 31/12/2013

Age groups	Actual cases in datasets	Estimated undiagnosed cases ^a^	Under estimation (%)	Prevalence^b^ (per 1000)	95% CI ^b^	Prevalence^b^ (per 1000) without undiagnosed cases	95% CI ^b^	RRT cases	RRT proportion (%)	RRT proportion with undiagnosed cases (%)
NT Aboriginal										
Male										
< 35	17	3	15.0	0.81	0.46–1.17	0.69	0.36–1.02	17	100.0	85.0
35–64	219	31	12.4	24.81	21.77–27.85	21.73	18.89–24.58	188	85.8	75.2
> 64	33	6	15.4	36.38	25.17–47.59	30.78	20.44–41.12	23	69.7	59.0
Aboriginal males	269	40	12.9	14.50	14.31–14.69	12.55	12.38–12.73	228	84.8	73.8
Female										
< 35	22	4	15.4	1.11	0.68–1.54	0.94	0.55–1.33	19	86.4	73.1
35–64	324	48	12.9	35.26	31.74–38.78	30.71	27.42–34.01	268	82.7	72.0
> 64	67	9	11.8	55.43	43.32–67.55	48.87	37.46–60.28	44	65.7	57.9
Aboriginal females	413	61	12.9	21.05	20.84–21.27	18.40	18.20–18.60	331	80.1	69.8
Aboriginal	682	101	12.9	17.97	17.82–18.11	15.65	15.52–15.79	559	82.0	71.4
NT non-Aboriginal										
Male										
< 35	12	2	14.3	0.30	0.14–0.45	0.25	0.11–0.40	12	100.0	85.7
35–64	37	6	14.0	1.12	0.79–1.46	0.97	0.66–1.28	33	89.2	76.7
> 64	26	4	13.3	4.24	2.73–5.75	3.67	2.26–5.08	20	76.9	66.7
Non-Aboriginal males	75	12	13.8	1.11	1.08–1.13	0.96	0.93–0.98	65	86.7	74.7
Female										
< 35	8	2	20.0	0.25	0.09–0.40	0.20	0.06–0.34	8	100.0	80.0
35–64	33	5	13.2	1.14	0.78–1.51	0.99	0.65–1.33	32	97.0	84.2
> 64	16	4	20.0	3.65	2.05–5.24	2.92	1.49–4.35	12	75.0	60.0
Non-Aboriginal females	57	11	16.2	1.02	0.99–1.05	0.85	0.82–0.87	52	91.2	76.5
Non-Aboriginal	132	23	14.8	1.07	1.05–1.09	0.91	0.89–0.92	117	88.6	75.5
Total	814	124	13.2	4.57	4.53–4.60	3.96	3.93–3.99	676	83.0	72.1

^a^ Capture-recapture model with 4 data sources for Aboriginal patients and 3 data sources for non-Aboriginal patients. ^b^ Age adjusted to 2001 Australian population

The results of the log-linear model for prevalence estimates are also presented in Table 2. In addition to 814 diagnosed (known) prevalent cases, there were estimated to be an additional 124 undiagnosed cases, with 101 in the Aboriginal and 23 in non-Aboriginal population. The overall proportion of underestimation was 12.9% for the Aboriginal population and 14.8% for the non-Aboriginal population. Including all cases, the ESKD prevalence in the total NT population was 3.9 per 1000 population, with 11.0 per 1000 Aboriginal population and 0.9 per 1000 non-Aboriginal population. After adjustment to the national age distribution, the age-adjusted prevalence was 4.6 for the NT total population, 18.0 per 1000 for the NT Aboriginal population, and 1.1 for the NT non-Aboriginal population (Table 2). The age-adjusted prevalence for females was higher than males in the NT Aboriginal population, while female and male rates were similar in the NT non-Aboriginal population. The age-specific prevalence rate ratios of NT Aboriginal to non-Aboriginal patients were 2.7 for males and 4.5 for females aged <35 years, 22.1 for males and 30.8 for females aged 35–64 years; 8.6 for males and 15.2 for females aged 65 years and over.

**Fig. 1:**
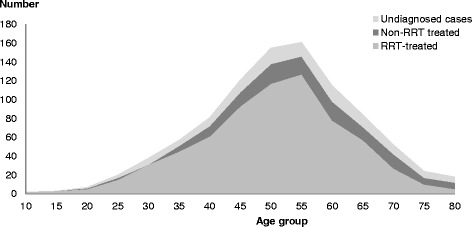
RRT treated, non-RRT treated and undiagnosed cases

As of 31 December 2013, there was an estimated total of 938 prevalent cases of whom 676 people were receiving RRT. The proportion of total ESKD cases receiving RRT treatment was 75.5% for non-Aboriginal and 71.4% for Aboriginal people (Table 2), much lower than the treatment proportions (89% and 81%) based on the observed prevalence. The proportion of patients receiving RRT treatment was higher in Aboriginal males than Aboriginal females with or without estimated undiagnosed cases for all age groups; the converse was true for non-Aboriginal patients. The age distribution of ESKD prevalent cases by type (RRT treated, non-RRT treated and undiagnosed cases) for the total NT population is presented in Fig. 1, and highlights the relative contribution of untreated and undiagnosed cases to total cases across age groups.

### Incidence

During 2013, there were 194 incident ESKD cases identified (Table 3); 161 (83.0%) were Aboriginal people. The log-linear model incidence estimates are presented in Table 3. In addition to 194 diagnosed cases there was an estimated 65 undiagnosed incident cases. The overall proportion of under estimation was 25.1%, with similar underestimation in Aboriginal (24.8%) and non-Aboriginal (26.7%) populations. The age-adjusted incidence for the total NT population was 1.35 cases per 1000 population, with a much higher age-adjusted incidence of 5.26 cases per 1000 population in the Aboriginal population than in the NT non-Aboriginal population (0.36 cases per 1000 population). The incidence among females was higher than among males in both NT Aboriginal and non-Aboriginal populations for all age groups. The highest incidence rate ratios of NT Aboriginal to non-Aboriginal were for the age group 35–64 years in both men and women (38.5 and 36.3 respectively).

**Table 3:** Incidence of end-stage kidney disease by age group, sex and Aboriginal status, Northern Territory, 2013

Age groups	Actual cases in datasets	Estimated undiagnosed cases ^a^	Under estimation (%)	Incidence ^b^ (per 1000)	95% CI ^b^	Incidence ^b^ (per 1000) without estimated undiagnosed cases	95% CI ^b^
NT Aboriginal							
Male							
< 35	4	3	42.9	0.28	0.07–0.50	0.16	0.00–0.32
35–64	55	16	22.5	7.05	5.41–8.68	5.46	4.02–6.90
> 64	13	5	27.8	16.79	9.10–24.48	12.13	5.57–18.68
Aboriginal males	72	24	25.0	4.95	3.77–6.14	3.70	2.68–4.72
Female							
< 35	6	3	33.3	0.38	0.13–0.63	0.26	0.05–0.46
35–64	64	20	23.8	7.96	6.27–9.66	6.07	4.59–7.55
> 64	19	6	24.0	18.23	11.15–25.32	13.86	7.67–20.05
Aboriginal females	89	29	24.6	5.54	4.40–6.68	4.20	3.20–5.19
Aboriginal	161	53	24.8	5.26	4.44–6.08	3.96	3.25–4.67
NT non-Aboriginal							
Male							
< 35	1	1	50.0	0.04	−0.02-0.10	0.02	−0.02-0.06
35–64	5	2	28.6	0.18	0.05–0.32	0.13	0.02–0.25
> 64	11	3	21.4	1.98	0.94–3.01	1.55	0.64–2.47
Non-Aboriginal males	17	6	26.1	0.34	0.19–0.49	0.26	0.13–0.38
Female							
< 35	3	1	25.0	0.11	0.01–0.21	0.07	−0.01-0.16
35–64	5	2	28.6	0.22	0.06–0.38	0.15	0.02–0.28
> 64	8	3	27.3	1.99	0.81–3.17	1.46	0.45–2.47
Non-Aboriginal females	16	6	27.3	0.39	0.22–0.56	0.28	0.13–0.42
Non-Aboriginal	33	12	26.7	0.36	0.25–0.47	0.27	0.17–0.36
Total	194	65	25.1	1.35	1.18–1.53	1.02	0.86–1.17

^a^ Capture-recapture model with 4 data sources for Aboriginal patients and 3 data sources for non-Aboriginal patients. ^b^ Age adjusted to 2001 Australian population

### Sensitivity analysis

The sensitivity analysis showed that a definition of ESKD using an eGFR cut-off of 7 ml/min/1.73 m2 resulted in a lower estimate of undiagnosed Aboriginal patients being identified in both incidence and prevalence models (Additional file 6: Table S6).

## Discussion

This study applied capture–recapture methods to unit level data linked across multiple data sources to estimate the number of ESKD cases in the NT Aboriginal and non-Aboriginal populations. This novel approach incorporates patients who are known to have either treated or untreated ESKD as well as an estimate for undiagnosed cases. Our study found age-adjusted prevalence of 18.0 and 1.1 per 1000 for the NT Aboriginal and non-Aboriginal population respectively, while the corresponding age-adjusted incidence was 5.26 and 0.36 per 1000 population. Most previous research has used participation rates for RRT as an indirect measure of the incidence of ESKD, however, not all people with ESKD will commence RRT [2, 4, 15, 22]. Our ESKD incidence estimates for total NT population of 1.02 per 1000 population however are also higher than the previous Australian study which linked ANZDATA data with national mortality data and estimated a total NT incidence of 0.72 per 1000 population [4]. The difference will be a combination of increased incidence since 2003–2007 and the addition, in our study, of an estimate for undiagnosed cases. Our study also confirms that the incidence of diagnosed ESKD for the NT Aboriginal population was much greater than national Aboriginal age-standardised estimates for the period 2009–2013 (3.96 vs 0.95 per 1000 population) [1]. Consistent with the previous report, our study reports earlier onset of ESKD for the Aboriginal population with a substantial excess among those aged 35–64 years, as well as a higher rate among females than males [2].

Estimated undiagnosed ESKD prevalent and incident Aboriginal cases were a much larger proportion (81% and 82% respectively) of all NT cases than the corresponding proportion of the total NT population (30%) [20], which is consistent with the high prevalence and incidence in the Aboriginal population [23–26]. The effect of different eGFR cut-off thresholds for the definition of ESKD on the number of undiagnosed Aboriginal cases was substantial. Using the threshold of an eGFR < 15 maintained consistency of a definition of ESKD across administrative and clinical datasets and followed international guidelines, whereas a threshold of an eGFR = <7 (based on the median eGFR at commencement of RRT in Australia) identified fewer patients. However, these differences did not alter a key outcome of the analysis: ESKD incidence and prevalence is much higher among Aboriginal than non-Aboriginal NT residents, even after accounting for undiagnosed cases.

All diagnosed patients had to have at least one contact with the health services to be found in one of the health datasets. Australia has a largely publicly funded health care system with universal access to health care. The national health care system allows Australians to access health care, regardless of their personal circumstances, while also giving options for individuals through private sector involvement in delivery and financing. Aboriginal Australians have much lower levels of private health insurance coverage compared to non-Aboriginal Australians (15% vs 51%), are more likely to use public hospital services and have lower rates of elective surgery [26]. Barriers to accessing care when needed vary between remote and non-remote areas, while 21% and 58% of NT Aboriginal population live in a remote or a very remote area [20, 27]. A previous NT study indicated that miscommunications and lack of shared understanding between Aboriginal patients and health staff in renal and hospital services often limited patients’ opportunities and capacities to make informed choices about their healthcare. Appropriate information and the associated understanding of the patients and their family are crucial for them to make the important decisions about treatment choice [28, 29].

While the overall prevalence estimates provide a broad measure for planning appropriate services, the incidence estimates are of even greater concern. An estimated 53 largely middle-aged Aboriginal incident ESKD cases were not identified in the health data, although the awareness of chronic kidney disease amongst clinicians and clinical coders is already high in the NT. This finding supports the need for further measures for timely diagnosis, to reduce premature death and improve access for suitable treatment. Such measures include improved support for health care providers to recognise ESKD, including a need for better risk stratification tools in the high-risk NT Aboriginal population.

A previous study reported that more Aboriginal females than Aboriginal males commence treatment for ESKD [30], which has been subsequently interpreted as NT Aboriginal women being more likely to start treatment [31]. Our results indicate that while NT Aboriginal women have a greater prevalence and incidence of ESKD than their male counterparts, they were less likely to access RRT. This finding should be the subject of further investigation. Overall, 28% of NT patients did not receive RRT treatment and the proportion of Aboriginal patients who did not receive RRT treatment (29%) was higher than the non-Aboriginal figure (24%). For each patient, the decision to commence RRT or to have conservative management (no RRT) is complex. Medically assessed suitability for RRT, prognosis, anticipated quality of life, treatment burden, accessibility of health services, and personal choice all play a part in the decision of whether or not to receive RRT treatment [1, 32]. ESKD has major medical and social implications for Aboriginal people [33]. Isolation, the need to travel and social dislocation are particular issues for Aboriginal patients from remote communities, which may deter some patients from undertaking RRT. Despite a significant increase since 1999 in the number of remote communities in which dialysis is provided [34], many areas of high need persist in having poor access to RRT service provision, with hundreds of kilometres between communities and the nearest dialysis unit [22, 35]. Local policies and clinical pathways to support patients who choose not to take up RRT treatment are being evaluated [32].

Our study has a number of limitations. First, it is possible that the linkage of the same individuals in different datasets was incomplete. This would have the effect of overestimating prevalence and incidence. However, the unique patient identifier within NT health data has previously been shown to be highly reliable, and a substantial majority of individuals within the samples were linked across data sources [11]. A second limitation is that capture-recapture methods assume a closed population. Although the NT Aboriginal population is highly mobile between communities, interstate net migration is minimal [36]. However, the non-Aboriginal population may move either into or out of the NT at a time of declining health or retirement. The similarity of non-Aboriginal results with the national estimate suggests that the effect of interstate migration may not be substantial [17]. A third limitation is that the study relied on recording of a clinical diagnosis in electronic data systems. Case notes and clinical reviews were not available to validate diagnoses. A fourth limitation is that the definitions for ESKD varied between data sources, for example between a laboratory diagnosis in a primary care setting and the commencement of maintenance dialysis leading to registry notification. Similarly not all patients with an eGFR <15 may need RRT. This variation means that an individual will have a diagnosis in one dataset but may not appear in a second dataset or the documentation may be delayed for several months. These differences will have little effect on the measurement of known cases but may result in an overestimation of undiagnosed cases, as shown in the sensitivity analysis. A final limitation is that only 5.5 years’ data were available for the study. If information had been available for a longer period, some additional cases may have been identified and apparent incident cases may have been recognised as having had an earlier onset. However, using four different data sources reduced the probability of having missed the index episode. Future estimates, based on these data sources, will benefit from the availability of additional years of data.

## Conclusions

This study demonstrates that data linkage, using existing administrative data sources, can be an efficient and accessible approach to informing a significant knowledge gap in renal services. Near-complete Aboriginal identification in NT health data sources also minimised the impact of misclassification on our findings [11]. Our study results highlight specific unmet needs in the comparatively young NT Aboriginal population. More generally in Australia, large-scale population studies are warranted in order to better understand the total burden, regional variation and social costs of ESKD. This information is necessary to develop strategies to reduce the total burden of ESKD and inform planning of comprehensive health service delivery in remote and very remote areas in Australia.

## Additional files

Additional file 1: Case definitions of End Stage Kidney Disease. (DOCX 17 kb)

Additional file 2: Number of incident ESKD cases in 4 data sources, Aboriginal population, Northern Territory 2013. (DOCX 17 kb)

Additional file 3: Number of incident ESKD cases in 3 data sources, non-Aboriginal population, Northern Territory 2013. (DOCX 16 kb)

Additional file 4: Number of prevalent ESKD cases in 4 data sources, Aboriginal population, Northern Territory 2013. (DOCX 18 kb)

Additional file 5: Number of prevalent ESKD cases in 3 data sources, non-Aboriginal population, Northern Territory 2013. (DOCX 16 kb)

Additional file 6: Sensitivity analysis for estimates of incidence and prevalence with varied eGFR cutpoints in Primary Care Information System data, by sex, and age group, Aboriginal population, Northern Territory 2013. (DOCX 20 kb)

## Abbreviations

ANZDATA: Australia and New Zealand Dialysis and Transplant Registry
BDM: Registry of Births, Deaths and Marriages
eGFR: Estimated glomerular filtration rate
ESKD: End stage kidney disease
HRN: Hospital Registration Number
HSD: Hospital Separations Dataset
NT: Northern Territory
PCIS: The Primary Care Information System
RRT: Renal replacement therapy

## References

[1] Australian Institute of Health and Welfare (2016). Incidence of end-stage kidney disease in Australia 1997–2013. (AIHW cat. No. PHE 211).

[2] Australia and New Zealand Dialysis and Transplant Registry (2016). 38th annual report.

[3] United States Renal Data System (2016). Chapter 13: international comparisons. 2016 USRDS annual data report: epidemiology of kidney disease in the United States.

[4] Australian Institute of Health and Welfare (2011). End-stage kidney disease in Australia: total incidence, 2003–2007 (AIHW cat. No. PHE 143).

[5] Krebs J (1989). Ecological methodology.

[6] Robles C, Marrett D, Clarke A, Risch A (1988). An application of capture-recapture methods to the estimation of completeness of cancer registration. J Clin Epidemiol.

[7] Yip P, Bruno G, Tajima N, Seber G, Buckland S, Cormack R (1995). Capture-recapture and multiple-record systems estimation I: history and theoretical development. Am J Epidemiol.

[8] Hook E, Regal R (1995). Capture-recapture methods in epidemiology: methods and limitations. Epidemiol Rev.

[9] Chao A, Tsay P, Lin SH, Shau WY, Chao DY (2001). The applications of capture-recapture models to epidemiological data. Stat Med.

[10] Foley M, Zhao Y, Condon J (2012). Demographic data quality assessment for northern territory public hospitals. 2011.

[11] Li SQ, Guthridge SL, Aratchige PE, Lowe MP, Wang Z, Zhao Y (2014). Dementia prevalence and incidence among the indigenous and non-indigenous populations of the northern territory. Med J Aust.

[12] Condon JR, Zhang X, Dempsey K, Garling L, Guthridge S (2016). Trends in cancer incidence and survival for indigenous and non-indigenous people in the northern territory. Med J Aust.

[13] Tay E, Li SQ, Guthridge S (2013). Mortality in the northern territory, 1967–2006.

[14] Colquhoun SM, Condon JR, Steer AC, Li SQ, Guthridge S, Carapetis JR (2015). Disparity in mortality from rheumatic heart disease in indigenous Australians. J Am Heart Assoc.

[15] Australian Institute of Health and Welfare (2014). Assessment of the coding of ESKD in deaths and hospitalisation data: a working paper using linked hospitalisation and deaths data from Western Australia and new South Wales (AIHW cat. No. PHE 182).

[16] You JQ, Lawton P, Zhao Y, Poppe S, Cameron N, Guthridge S (2015). Renal replacement therapy demand study, northern territory, 2001 to 2022.

[17] Sparke C, Moon L, Green F, Mathew T, Cass A, Chadban S (2013). Estimating the total incidence of kidney failure in Australia including individuals who are not treated by dialysis or transplantation. Am J Kidney Dis.

[18] WONCA International Classification Committee (2005). International classification of primary care. Revised 2nd ed.

[19] Kidney Health Australia (2012). Chronic kidney disease (CKD) Management in General Practice.

[20] Australian Bureau of Statistics (2016). Australian demographic statistics (ABS cat. No. 3101.0.).

[21] Australian Bureau of Statistics (2013). 31010DO003_201212 standard population for use in age-standardisation.

[22] Preston-Thomas A, Cass A, O'Rourke P (2007). Trends in the incidence of treated end-stage kidney disease among indigenous Australians and access to treatment. ANZJPH.

[23] Australian Institute of Health and Welfare (2011). Chronic kidney disease in aboriginal and Torres Strait islander people 2011 (AIHW cat. No. PHE 151).

[24] Byron P, Zhao Y, Guthridge S, Brailsford R, Stacey F, Parkinson J (2005). Medicare and pharmaceutical benefits scheme usage patterns in the northern territory 1993/94 to 2003/04.

[25] Australian Health Ministers’ Advisory Council (2015). Aboriginal and Torres Strait islander health performance framework 2014 report.

[26] Pulver LJ, Haswell MR, Ring I, Waldon J, Clark W, Whetung V (2010). Indigenous health–Australia, Canada, Aotearoa New Zealand and the United States-laying claim to a future that embraces health for us all.

[27] Australian Institute of Health and Welfare (2015). Aboriginal and Torres Strait islander health performance framework 2014 report: northern territory (AIHW cat. No. IHW 159).

[28] Coulehan K, Brown I, Christie M, Gorham G, Lowell A, Marrnanyin B (2005). Sharing the true stories: evaluating strategies to improve communication between health staff and aboriginal patients, stage 2 report.

[29] Anderson K, Devitt J, Cunningham J, Preece C, Cass A (2008). “All they said was my kidneys were dead”: indigenous Australian patients’ understanding of their chronic kidney disease. Med J Aust.

[30] McDonald P, Russ R (2003). Current incidence, treatment patterns and outcome of end-stage renal disease among indigenous groups in Australia and New Zealand. Nephrology.

[31] Australian Institute of Health and Welfare (2012). Dialysis and kidney transplantation in Australia: 1991–2010. (AIHW cat. No. PHE 162).

[32] Morton RL, Snelling P, Webster AC, Rose J, Masterson R, Johnson DW (2012). Factors influencing patient choice of dialysis versus conservative care to treat end-stage kidney disease. CMAJ.

[33] Anderson K, Cunningham J, Devitt J, Cass A (2013). The IMPAKT study: using qualitative research to explore the impact of end-stage kidney disease and its treatments on aboriginal and Torres Strait islander Australians. Kidney Int Suppl.

[34] Gorham G, Wagner L, Jose M (2005). The northern Territory’s remote and community-based haemodialysis program: interesting times. Ren Soc Aust J.

[35] Gorham G (2003). Prevention and treatment options for renal disease in the northern territory (with particular reference to the Barkly region).

[36] Condon JR, Barnes T, Cunningham J, Smith L (2004). Demographic characteristics and trends of the northern territory indigenous population, 1966 to 2001. Occasional paper.

