# Supplementary material for: Estimating the total prevalence and incidence of end-stage kidney disease among Aboriginal and non-Aboriginal populations in the Northern Territory of Australia, using multiple data sources
Source: BMC Nephrol. 2018 Jan 15;19:15. doi: 10.1186/s12882-017-0791-3 (PMC5769509; doi:10.1186/s12882-017-0791-3)
Supplement: Supplementary file 1 — Case definitions of End Stage Kidney Disease. (DOCX 17 kb) [file 12882_2017_791_MOESM1_ESM.docx]

**Additional file 1: Case definitions of End Stage Kidney Disease**

| **Hospital Separation Data (HSD): Principal or additional diagnosis codes** | |
| --- | --- |
| **ICD-10-AM codes** | **Definition** |
| N18.5 (ICD-10-AM 6th edition) | Chronic kidney disease—stage 5 |
| T82.4 | Mechanical complication of vascular dialysis catheter |
| T86.1 | Kidney transplant failure and rejection |
| Z49 | Care involving dialysis |
| Z94.0 | Kidney transplant status |
| Z99.2 | Dependence on kidney dialysis |
| **Australia and New Zealand Dialysis and Transplant Registry (ANZDATA)** | |
| All cases | Routine chronic dialysis or a kidney transplant |
| **Primary Care Information System (PCIS)** | |
| **ICPC codes** | **Definition** |
| U59001, U59007, U59008, U59009, U59J99 | Dialysis |
| U28001 | Renal transplant |
| U88J95, U99039 | Stage 5 chronic kidney disease |
| Laboratory | A glomerular filtration rate (eGFR) of less than <15 mL/min/1.73m2 with previous eGFR <60 mL/min/1.73m2 at least three months prior |
| **Birth Deaths and Marriages Registry (BDM) — leading or other cause of death** | |
| Leading cause of death | Dialysis |
|  | renal transplant |
|  | end-stage kidney (renal) disease |
|  | end-stage kidney (renal) failure |
|  | chronic kidney (renal) failure |
| Other causes of death | End-stage kidney (renal) failure |
