# Supplementary material for: Estimating the total prevalence and incidence of end-stage kidney disease among Aboriginal and non-Aboriginal populations in the Northern Territory of Australia, using multiple data sources
Source: BMC Nephrol. 2018 Jan 15;19:15. doi: 10.1186/s12882-017-0791-3 (PMC5769509; doi:10.1186/s12882-017-0791-3)
Supplement: Supplementary file 3 — Number of incident ESKD cases in 3 data sources, non-Aboriginal population, Northern Territory 2013. (DOCX 16 kb) [file 12882_2017_791_MOESM3_ESM.docx]

**Additional file 3: Number of incident ESKD cases in 3 data sources, non-Aboriginal population, Northern Territory 2013**

| **ANZDATA** | **HSD** | **BDM** | **Frequency** |
| --- | --- | --- | --- |
| No | No | No | 12* |
|  |  | Yes | 3 |
|  | Yes | No | 23 |
|  |  | Yes | 0 |
| Yes | No | No | 0 |
|  |  | Yes | 0 |
|  | Yes | No | 7 |
|  |  | Yes | 0 |
| Total |  |  | 33 |

*Estimated number from a log-linear capture recapture model based on the number of patients in the HSD, ANZDATA and death registration datasets.
