# Supplementary material for: Estimating the total prevalence and incidence of end-stage kidney disease among Aboriginal and non-Aboriginal populations in the Northern Territory of Australia, using multiple data sources
Source: BMC Nephrol. 2018 Jan 15;19:15. doi: 10.1186/s12882-017-0791-3 (PMC5769509; doi:10.1186/s12882-017-0791-3)
Supplement: Supplementary file 6 — Sensitivity analysis for estimates of incidence and prevalence with varied eGFR cutpoints in Primary Care Information System data, by sex, and age group, Aboriginal population, Northern Territory 2013. (DOCX 20 kb) [file 12882_2017_791_MOESM6_ESM.docx]

**Additional file 6: Sensitivity analysis for estimates of incidence and prevalence with varied eGFR cutpoints in Primary Care Information System data, by sex, and age group, Aboriginal population, Northern Territory 2013**

| **Incidence** | | | | | | |
| --- | --- | --- | --- | --- | --- | --- |
|  | **eGFR<15** | | |  | **eGFR=<7** | |
| **Age groups** | **Actual cases in datasets** | | **Estimated undiagnosed cases** |  | **Actual cases in datasets** | **Estimated undiagnosed cases** |
| **NT Aboriginal** |  | |  |  |  |  |
| **Male** |  | |  |  |  |  |
| <35 | 4 | | 3 |  | 4 | 1 |
| 35-64 | 55 | | 16 |  | 52 | 8 |
| >64 | 13 | | 5 |  | 13 | 2 |
| **Aboriginal males** | **72** | | **24** |  | **69** | **11** |
| **Female** |  | |  |  |  |  |
| <35 | 6 | | 3 |  | 6 | 2 |
| 35-64 | 64 | | 20 |  | 61 | 10 |
| >64 | 19 | | 6 |  | 16 | 3 |
| **Aboriginal females** | **89** | | **29** |  | **83** | **15** |
| **Aboriginal** | **161** | | **53** |  | **152** | **26** |
| **Prevalence** | | | | | | |
|  | | **eGFR<15** | |  | **eGFR=<7** | |
| **Age groups** | | **Actual cases in datasets** | **Estimated undiagnosed cases** |  | **Actual cases in datasets** | **Estimated undiagnosed cases** |
| **NT Aboriginal** | |  |  |  |  |  |
| **Male** | |  |  |  |  |  |
| <35 | | 17 | 3 |  | 17 | 2 |
| 35-64 | | 219 | 31 |  | 217 | 20 |
| >64 | | 33 | 6 |  | 32 | 4 |
| **Aboriginal males** | | **269** | **40** |  | **266** | **26** |
| **Female** | |  |  |  |  |  |
| <35 | | 22 | 4 |  | 22 | 3 |
| 35-64 | | 324 | 48 |  | 316 | 30 |
| >64 | | 67 | 9 |  | 64 | 6 |
| **Aboriginal females** | | **413** | **61** |  | **402** | **39** |
| **Aboriginal** | | **682** | **101** |  | **668** | **65** |
